# Pathological complete response to perioperative treatment with darolutamide plus ADT in locally advanced prostate cancer without PTEN or RB1 loss: a case report

**DOI:** 10.3389/fphar.2026.1829605

**Published:** 2026-06-03

**Authors:** Zhihui Zhang, Jiwei Zhai, Qimei Ma, Yuzhu Xiang, Jiajun Kan, Junhao Chu, Chunxiao Wei, Muwen Wang

**Affiliations:** 1 Department of Urology, Shandong Provincial Hospital Affiliated to Shandong First Medical University, Jinan, China; 2 Central Sterile Supply Department, Shandong Provincial Hospital Affiliated to Shandong First Medical University, Jinan, China

**Keywords:** androgen deprivation therapy, case report, darolutamide, locally advanced prostate cancer, pathological complete response, pten, Rb1

## Abstract

Locally advanced prostate cancer (LAPC) is associated with a higher risk of recurrence and metastasis. Perioperative intensified systemic therapy may contribute to tumor downstaging. However, clinical evidence supporting darolutamide-based treatment in LAPC remains limited, and biomarker-informed treatment in this setting is not well established. We report the case of a 70-year-old man with cT4N1M0 LAPC without PTEN or RB1 loss by immunohistochemistry (IHC). At presentation, the patient’s total prostate-specific antigen (TPSA) level was 88.80 ng/mL. Prostate multiparametric magnetic resonance imaging (mpMRI) revealed the invasion of right lateral bladder wall, bladder neck and bilateral seminal vesicles, and enlarged pelvic lymph nodes. Conventional imaging, including bone scintigraphy and computed tomography showed no evidence of distant metastasis. Transperineal prostate biopsy confirmed prostatic acinar adenocarcinoma with a Gleason score of 5 + 4 = 9 (ISUP Grade Group 5). The initial intensified systemic regimen is the doublet therapy of Darolutamide (600 mg orally twice daily) and goserelin (10.8 mg administered subcutaneously every 3 months). Castration-level testosterone was achieved after 1 month, and TPSA decreased sharply from 88.80 ng/mL to 0.15 ng/mL at month 1 and 0.008 ng/mL at month 8. After 8 months of doublet therapy, follow-up mpMRI showed no residual suspicious lesion or enlarged pelvic lymph nodes. As a tailored local consolidation strategy, the patient underwent laparoscopic radical prostatectomy (LRP) and pelvic lymph node dissection after MDT discussion. A pathological complete response (pCR) was achieved, as confirmed by postoperative pathology showing no residual tumor in the prostatectomy specimen or pelvic lymph nodes (ypT0N0). The recovery of urinary incontinence was achieved after 2 months following LRP, while Darolutamide plus ADT was continued postoperatively. During 20 months of follow-up, the patient maintained an undetectable PSA, and had no radiographic evidence of disease relapse with well treatment tolerance. This case suggests that darolutamide plus ADT, administered as a tailored perioperative intensified systemic strategy, may induce a rapid and profound PSA response and may be associated with pCR in selected patients with very high-risk LAPC, while further investigation is warranted.

## 1 Introduction

Prostate cancer (PCa) predominantly affects older men, and nearly 15% of patients present with high-risk and/or locally advanced disease at diagnosis (Freedland et al., 2025; Raychaudhuri et al., 2025). Locally advanced prostate cancer (LAPC), generally defined as cT3–4 disease and/or regional lymph node involvement in the absence of distant metastasis, is characterized by extensive local invasion and a persistently high risk of recurrence and distant progression even after definitive treatment (Van den Broeck et al., 2020; Kafka and Heidegger, 2026). Imaging evidence of bladder involvement or seminal vesicle invasion often reflects aggressive local disease and underscores the need for optimized multimodal treatment strategies (Xie et al., 2017; Rebello et al., 2021). Current major international guidelines, including those from the National Comprehensive Cancer Network and the European Association of Urology, recommend definitive radiotherapy plus long-term androgen deprivation therapy (ADT) as a standard curative-intent option for LAPC, while radical prostatectomy with pelvic lymph node dissection can be considered in appropriately selected patients as part of a multimodal treatment strategy (Cornford et al., 2026; National Comprehensive Cancer Network, 2026). In parallel, androgen receptor pathway inhibitors (ARPIs), including abiraterone and enzalutamide, have been investigated as treatment intensification strategies in selected high-risk patients, while darolutamide has demonstrated robust clinical benefits in defined disease settings such as non-metastatic castration-resistant prostate cancer (nmCRPC) and metastatic hormone-sensitive prostate cancer (mHSPC) (Smith et al., 2018; Fizazi et al., 2019; Smith et al., 2022). Given the molecular heterogeneity of PCa and the established association of PTEN and RB1 alterations with aggressive tumor behavior, treatment resistance, and adverse clinical outcomes (Wise et al., 2017), high-quality prospective evidence supporting biomarker-informed treatment selection in LAPC remains limited, and clinical data on darolutamide as an intensified systemic option for LAPC are still insufficient.

Therefore, clarifying the clinical context in which ARPI-based intensified systemic therapy may produce profound responses remains clinically relevant, particularly in patients without PTEN or RB1 loss. In this report, we describe a patient with cT4N1M0 LAPC without PTEN or RB1 loss who received darolutamide plus ADT as initial intensified doublet therapy, with subsequent treatment tailored according to biochemical and radiographic responses. After a rapid PSA decline accompanied by marked radiographic response, laparoscopic radical prostatectomy (LRP) with PLND was performed as a tailored local consolidation strategy after multidisciplinary team (MDT) evaluation. Postoperative pathological examination confirmed ypT0N0 disease, indicating pathological complete response (pCR). This report summarizes the diagnostic and tailored treatment, as well as the follow-up outcome, of this case and aims to provide a clinical reference for the management of selected patients with LAPC.

## 2 Case report

### 2.1 Patient information

**FIGURE 1:**
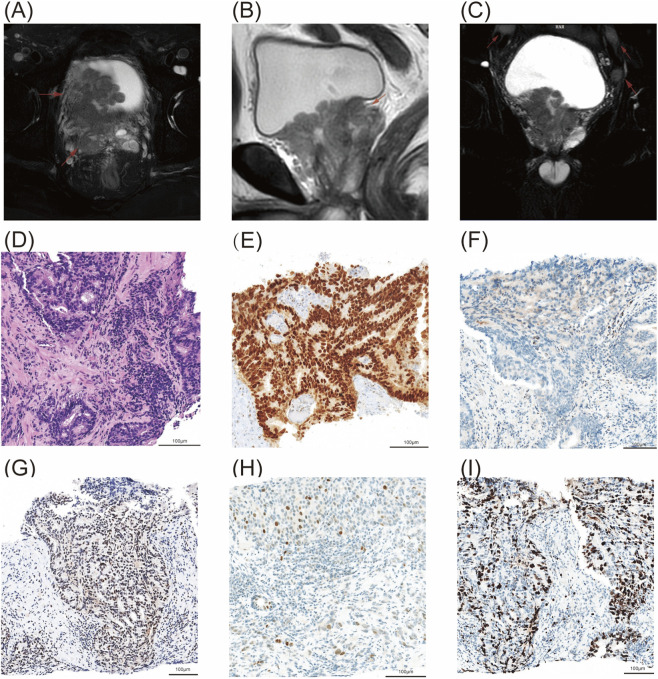
Imaging findings and pathological features of the prostate needle biopsy at initial diagnosis. **(A–C)** Prostate multiparametric magnetic resonance imaging showed a prostatic tumor involving the right lateral bladder wall, bladder neck, and bilateral seminal vesicles **(A, B)**, with multiple enlarged pelvic lymph nodes **(C)**. **(D–I)** Transperineal prostate needle biopsy. Hematoxylin and eosin (HE) staining revealed prostatic acinar adenocarcinoma with a Gleason score of 5 + 4 = 9 (ISUP Grade Group 5) **(D)**. Immunohistochemistry demonstrated positive staining for AR **(E)**, PTEN **(F)**, RB1 **(G)**, p53 **(H)**, and Ki-67 (50%) **(I)**.

A 70-year-old man presented to Shandong Provincial Hospital, affiliated with Shandong First Medical University, in October 2023 with a 6-month history of progressively worsening dysuria. On presentation, his Eastern Cooperative Oncology Group (ECOG) performance status was 0. He had no significant comorbidities, including hypertension, coronary artery disease, or diabetes mellitus. He denied any prior history of prostate-related disease. There was no known family history of prostate cancer or other malignancies. Digital rectal examination revealed diffuse induration of the posterior aspect of the prostate, with an irregular surface and limited mobility. Laboratory evaluation showed a serum total prostate-specific antigen (TPSA) level of 88.80 ng/mL, while routine hematologic parameters and liver and renal function tests were within normal limits. Prostate multiparametric magnetic resonance imaging (mpMRI) demonstrated locally advanced disease, with tumor invasion of adjacent structures, including the right lateral bladder wall, bladder neck, and bilateral seminal vesicles, as well as multiple enlarged pelvic lymph nodes (Figures 1A–C). Conventional imaging assessments, including bone scintigraphy, chest and abdominal computed tomography showed no definite evidence of distant metastasis. Although further prostate-specific membrane antigen positron emission tomography/computed tomography (PSMA PET/CT) was recommended for more accurate staging, the patient declined this examination. After informed consent had been obtained from the patient and his family, an ultrasound-guided transperineal prostate needle biopsy involving six cores was performed under local anesthesia on 26 October 2023. Histopathological examination showed that all six biopsy cores were positive for prostatic acinar adenocarcinoma, with a Gleason score of 5 + 4 = 9 (ISUP Grade Group 5) (Figure 1D). IHC results were interpreted by pathologists according to staining presence or loss and staining patterns in tumor cells. IHC demonstrated positive AR expression, no PTEN or RB1 loss, and a wild-type p53 staining pattern (Figures 1E–I). The Ki-67 labeling index was 50%, estimated as the percentage of positively stained tumor nuclei in hotspot areas. Based on the imaging and pathological findings and according to the 8th edition of the AJCC TNM staging system, the patient was diagnosed with cT4N1M0 LAPC, without PTEN or RB1 loss.

### 2.2 Course of treatment

Following MDT evaluation involving urology, medical oncology, radiation oncology, radiology, and pathology, radiotherapy-based multimodal treatment combined with long-term ADT was considered an established standard-of-care option. However, the patient declined radiotherapy because of concerns about radiation-related complications, particularly hemorrhagic radiation-induced cystitis or colitis. Given the extensive local invasion at presentation and concerns regarding residual disease involving the right lateral bladder wall and bladder neck, as well as the potential risk of tumor dissemination, radical surgery was not planned at the outset. Therefore, darolutamide plus ADT was initiated in October 2023 as an intensified systemic treatment strategy. The regimen consisted of darolutamide 600 mg orally twice daily and goserelin 10.8 mg administered subcutaneously every 3 months.

**FIGURE 2:**
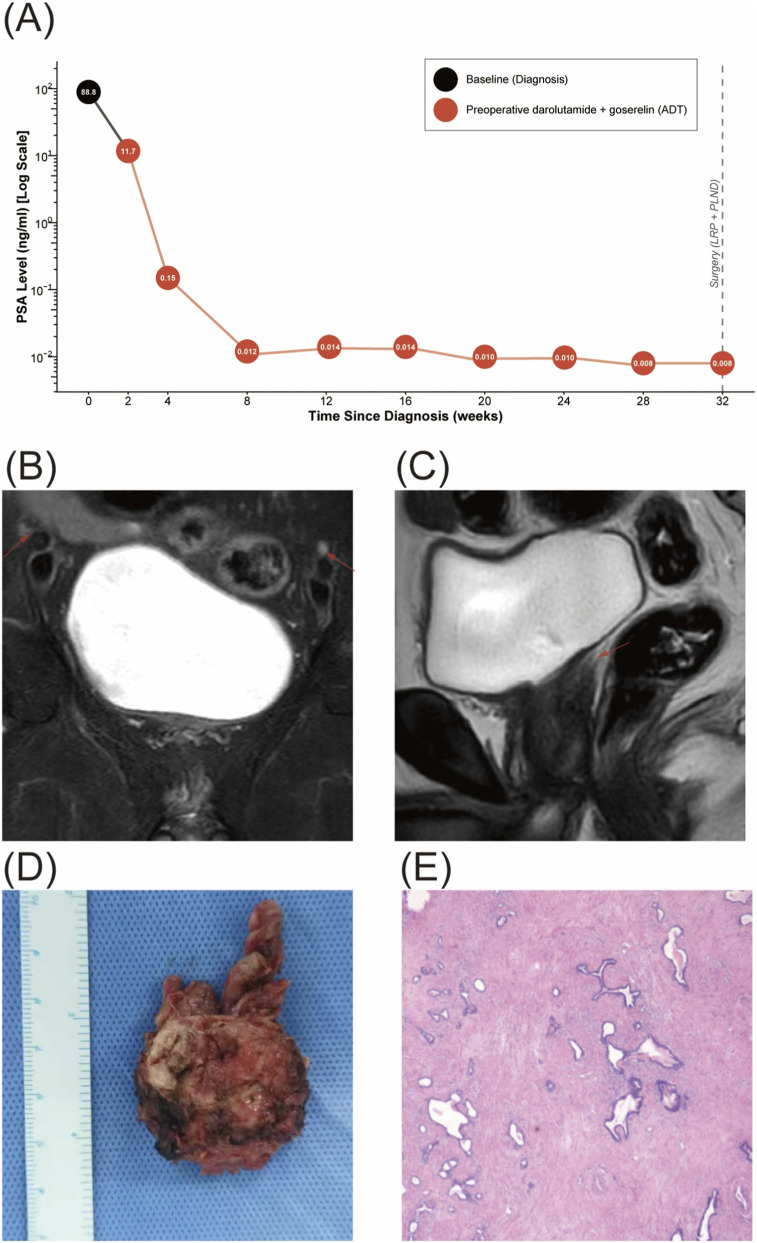
Treatment response during preoperative darolutamide plus ADT and postoperative pathological findings. **(A)** Dynamic changes in TPSA during the 8-month preoperative darolutamide plus ADT treatment period. **(B,C)** Multiparametric MRI after 8 months of treatment showed no residual suspicious lesion. **(D)** Gross specimen obtained after laparoscopic radical prostatectomy. **(E)** Postoperative pathological examination showed fibrosis and chronic inflammatory cell infiltration, with no residual tumor cells identified.

At 1 month after treatment initiation, serum testosterone decreased to 8.5 ng/dL, consistent with castrate testosterone levels (<50 ng/dL), and remained at castrate levels thereafter. As shown in Figure 2A, TPSA declined rapidly from baseline to 0.15 ng/mL at month 1, 0.012 ng/mL at month 2, and 0.008 ng/mL at month 8, remaining at an extremely low level throughout the treatment course. Follow-up mpMRI performed after 8 months of treatment demonstrated no definite residual lesion in the prostate, regression of the previously noted right lateral bladder wall, bladder neck, and seminal vesicle involvement, and no definite pelvic lymphadenopathy (Figures 2B,C). After 8 months of treatment, the sustained biochemical response and significant radiographic regression prompted repeat MDT evaluation. After discussion with the patient and his family, LRP with PLND was performed in June 2024 as a tailored local consolidation strategy based on the updated MDT recommendation. The operative time was approximately 3 h, with an estimated blood loss of 100 mL, and no intraoperative or perioperative complications occurred. Intraoperatively, mild adhesions were observed between the prostate and surrounding tissues, and the prostate, seminal vesicles, and bilateral vas deferens ampullae were completely resected (Figure 2D). Extensive sampling of the postoperative specimen revealed treatment-related changes, including fibrosis and chronic inflammatory cell infiltration, with no residual tumor cells identified on microscopic examination (Figure 2E). No metastatic involvement was identified in the dissected lymph nodes. Accordingly, the postoperative pathological findings showed no residual tumor in the prostatectomy specimen or dissected pelvic lymph nodes (ypT0N0), consistent with pCR.

### 2.3 Follow-up

**FIGURE 3:**
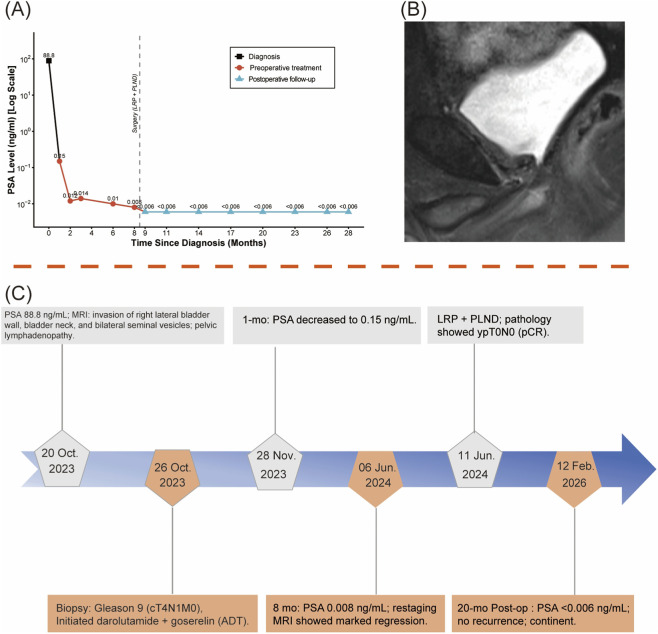
Follow-up outcomes and treatment timeline. **(A)** Dynamic changes in TPSA during the perioperative period and follow-up. **(B)** Pelvic MRI performed 20 months after surgery showed no evidence of recurrence. **(C)** Schematic timeline of the diagnostic and treatment course.

Given the patient’s very-high-risk PCa at presentation and substantial risk of recurrence, personalized adjuvant therapy with darolutamide plus goserelin was continued after LRP. The patient regained satisfactory urinary continence by 2 months postoperatively, and PSA remained undetectable (<0.006 ng/mL) during 20 months of follow-up through February 2026 (Figure 3A). Follow-up pelvic MRI performed approximately 20 months postoperatively showed no evidence of local recurrence or pelvic lymphadenopathy (Figure 3B). During treatment, the patient experienced no apparent rash or fatigue, liver and renal function tests remained within normal limits, and no dose reduction or treatment discontinuation was required due to adverse events. The patient also reported satisfaction with the treatment strategy. A schematic timeline of the diagnostic and treatment course is provided in Figure 3C.

## 3 Discussion

This case describes the achievement of pCR in a patient with very-high-risk LAPC without PTEN or RB1 loss, who was initially staged as cT4N1M0 and managed using a personalized darolutamide-based perioperative treatment strategy. As surgery was not initially planned because of bladder invasion by PCa, darolutamide plus ADT was first administered as initial intensified doublet therapy, with the subsequent treatment strategy adjusted according to marked biochemical and radiographic responses. After 8 months of treatment, the patient achieved sustained PSA suppression and significant radiographic regression, prompting LRP with PLND as a tailored local consolidation strategy based on the updated MDT recommendation. Postoperative pathology after LRP with PLND demonstrated ypT0N0 disease, consistent with pCR. After LRP with PLND, adjuvant therapy with darolutamide plus ADT was continued, and during 20 months of postoperative follow-up, PSA remained undetectable, with no radiographic evidence of recurrence.

LAPC, particularly when presenting with adjacent organ invasion or regional lymph node involvement, is associated with an increased risk of recurrence and distant metastasis and poses greater challenges to both local and systemic disease control. Therefore, management usually requires a tailored multimodal strategy, combining systemic therapy with consolidative local treatment (Kishan et al., 2022). For clinically node-positive LAPC, current guidelines recommend radiotherapy-based multimodal treatment with long-term ADT, with abiraterone recommended as a preferred treatment intensification option. Radical prostatectomy may also be considered in selected patients (Cornford et al., 2026; National Comprehensive Cancer Network, 2026). The clinical benefit of radiotherapy in LAPC or high-risk prostate cancer has been demonstrated in landmark randomized trials. In the SPCG-7/SFUO-3 trial, adding local radiotherapy to endocrine therapy approximately halved 10-year prostate cancer-specific mortality and substantially reduced overall mortality compared with endocrine therapy alone (Widmark et al., 2009). Similarly, the NCIC CTG PR.3/MRC PR07 trial showed that adding radiotherapy to ADT improved overall survival in patients with LAPC (Mason et al., 2015). Modern image-guided radiotherapy techniques have improved treatment precision and may enhance the therapeutic ratio, thereby supporting their important role in local disease control (Wang et al., 2023). Nevertheless, radiotherapy may still be associated with genitourinary and gastrointestinal toxicity (Matta et al., 2019), which can influence patient preference and shared decision-making.

In recent years, intensified systemic therapy with ADT plus an ARPI has been increasingly explored in the preoperative or perioperative setting for high-risk localized prostate cancer and LAPC. Previous phase II studies incorporating abiraterone or enzalutamide have suggested reduced residual tumor burden and improved pathological downstaging in selected patients, although pCR remains uncommon and the long-term oncologic benefit remains uncertain (Taplin et al., 2014; Montgomery et al., 2017). Postoperative ARPI-based intensification is also being explored. In the phase II Apa-RP study, adjuvant apalutamide plus ADT after radical prostatectomy achieved a 24-month confirmed biochemical recurrence-free rate of 100% (Shore et al., 2024). In the present case, radiotherapy plus long-term ADT was recommended following MDT evaluation, but the patient declined radiotherapy because of concerns regarding radiation-related complications particularly hemorrhagic radiation-induced cystitis or colitis. Given the extensive local invasion and concerns about tumor extension to the right lateral bladder wall and bladder neck, radical surgery was not planned at the outset. Therefore, darolutamide plus ADT was selected as a tailored initial intensified systemic doublet therapy.

Darolutamide is a second-generation androgen receptor pathway inhibitor that suppresses AR signaling through high-affinity binding to the AR ligand-binding domain (Fizazi et al., 2018). The ARAMIS trial, its subsequent overall survival analysis, and the ARASENS trial, all published in *The New England Journal of Medicine*, have demonstrated clear clinical benefits of darolutamide in patients with nmCRPC and mHSPC (Fizazi et al., 2019; Fizazi et al., 2020; Smith et al., 2022), thereby providing a strong evidence base for its further investigation in the perioperative setting. Compared with other ARPIs, darolutamide has a distinct molecular structure, and its major active metabolite, keto-darolutamide, also exhibits potent AR antagonistic activity (Sugawara et al., 2019). Previous studies have shown that darolutamide retains antagonistic activity against a range of clinically relevant AR mutants, suggesting potential advantages in selected contexts of AR alteration–associated treatment resistance (Sugawara et al., 2019; Lallous et al., 2021). In addition, darolutamide is characterized by low central nervous system penetration, limited potential for drug–drug interactions, and an overall favorable tolerability profile (Crawford et al., 2020). Mechanistically, goserelin, a luteinizing hormone-releasing hormone (LHRH) agonist, reduces circulating androgen levels by suppressing testicular testosterone production (Chrisp and Goa, 1991), whereas darolutamide directly inhibits AR signaling through receptor-level blockade. Together, these agents may achieve dual suppression of the androgen axis. In tumors that remain predominantly AR-driven, this intensified blockade may provide a biological basis for deeper suppression of AR signaling and may be compatible with the favorable biochemical and radiographic response observed in this case. Taken together, these pharmacological characteristics, along with the patient’s locally advanced disease and the need to consider long-term treatment tolerability, supported the use of darolutamide plus ADT as an initial intensified systemic treatment strategy.

At the molecular level, alterations in tumor suppressor genes (TSGs), particularly PTEN, TP53, and RB1, are frequent in prostate cancer and are associated with aggressive tumor biology, treatment resistance, and adverse clinical outcomes (Hamid et al., 2019). PTEN, a key negative regulator of the PI3K-AKT pathway, has been linked to tumor progression and adverse clinical outcomes, whereas RB1 loss is associated with dedifferentiation, lineage plasticity, and resistance to AR-directed therapy (Mu et al., 2017; Wise et al., 2017; Nyquist et al., 2020). TP53 abnormalities also reflect aggressive disease biology and poor prognosis (Maddah et al., 2024). Mechanistically, PTEN loss may attenuate dependence on androgen receptor (AR) signaling by activating PI3K-AKT bypass signaling (Carver et al., 2011), whereas RB1 loss has been linked to lineage plasticity, transition toward a less AR-dependent or AR-indifferent state, and resistance to AR-directed therapy, particularly when accompanied by TP53 loss (Ku et al., 2017). In this context, the absence of PTEN or RB1 loss on IHC in the present case may be biologically consistent with preserved AR dependence and a lower propensity for lineage plasticity, providing a plausible biological context for the favorable response observed in this case. However, this observation should be interpreted cautiously and should not be taken as evidence that the absence of PTEN or RB1 loss on IHC directly predicted treatment sensitivity in this particular patient.

This report has several limitations. First, next-generation sequencing was not performed. Although IHC showed no evidence of PTEN or RB1 loss, IHC reflects protein-level expression patterns rather than definitive genomic status and therefore cannot exclude underlying gene deletion, mutation, or copy-number alterations. Second, PSMA PET/CT was not performed for initial staging. Although conventional imaging showed no definite evidence of distant metastasis, occult metastatic disease cannot be completely excluded given the patient’s very-high-risk features; therefore, the initial M0 classification and pathological response should be interpreted cautiously. Third, the treatment strategy in this case was personalized rather than protocol-defined or guideline-established. Since the patient was ineligible for docetaxel, darolutamide plus ADT was ultimately administered as an intensified systemic doublet strategy, and the subsequent treatment strategy was adjusted according to follow-up biochemical and radiographic assessments. Surgery was not planned as an initial treatment option; therefore, the preoperative treatment duration, choice of local consolidation surgery, and postoperative continuation of darolutamide plus ADT were all individualized decisions. The optimal treatment duration, timing of surgery, and postoperative maintenance strategy remain uncertain. In addition, although no recurrence was observed during 20 months of postoperative follow-up, longer observation is required to assess durable oncologic control and potential long-term survival benefit.

## 4 Conclusion

In summary, LAPC is characterized by extension to adjacent organs and an increased risk of recurrence, and its management generally requires MDT-based decision-making that integrates systemic therapy with definitive local treatment. In the present case, a patient with very-high-risk cT4N1M0 LAPC without PTEN or RB1 loss received darolutamide plus ADT as initial intensified systemic therapy within a tailored perioperative strategy, achieved a rapid and profound PSA response, and ultimately attained pCR after local consolidation surgery. This case suggests that darolutamide-based intensified systemic therapy may have potential value as part of a tailored perioperative strategy in selected patients with LAPC. Further prospective studies with larger cohorts and longer follow-up are needed.

## Data Availability

The original contributions presented in the study are included in the article/supplementary material, further inquiries can be directed to the corresponding authors.
